# Brazilian Stingless Bee Geopropolis Exhibit Antioxidant Properties and Anticancer Potential Against Hepatocellular Carcinoma Cells

**DOI:** 10.3390/antiox14020141

**Published:** 2025-01-24

**Authors:** Mariana Muniz da Paz, Kamila Marques Sette, Raissa Eduardo dos Santos, Ana Luiza Barbosa e Vasconcelos, Danielly C. Ferraz da Costa, Ana Claudia F. Amaral, Igor Almeida Rodrigues, Luciana Pereira Rangel

**Affiliations:** 1 Programa de Pós Graduação em Ciências Farmacêuticas, Faculdade de Farmácia, Universidade Federal do Rio de Janeiro, Rio de Janeiro 21941-902, RJ, Brazil; 2 Instituto de Nutrição, Universidade do Estado do Rio de Janeiro, Rio de Janeiro 20550-013, RJ, Brazil; 3 Farmanguinhos, Fundação Oswaldo Cruz, Rio de Janeiro 21041-000, RJ, Brazil; 4 Faculdade de Farmácia, Universidade Federal do Rio de Janeiro, Rio de Janeiro 21941-902, RJ, Brazil

**Keywords:** *Melipona bicolor*, *Melipona marginata*, *Melipona mondury*, hepatocellular carcinoma, antioxidant, anticancer

## Abstract

Hepatocellular carcinoma (HCC) is the third most common cancer in terms of mortality and the sixth in incidence worldwide. Treatment varies by tumor stage, but low survival rates are common across all stages. Due to these poor outcomes, there is a critical need for new treatment options and lead compounds, prompting an active search. Geopropolis has been identified as a source of bioactive compounds with various pharmacological activities, including anticancer effects against different types of cancer. Since stingless bees may be selective for native botanical species, the geopropolis they produce can have an unusual chemical profile. In this study, we report the antioxidant properties and anticancer potential of geopropolis extracts produced by *Melipona bicolor*, *M. marginata*, and *M. mondury* using 2D- and 3D- cell culture models. The chemical profile of these samples using UPLC-QTOF HRMS/MS indicated ferreirin and dihydrokaempferide as the main flavonoids, along with cupressic acid and 15-acetoxyisocupressic acid as the most abundant diterpenoids. Interestingly, artepillin C, a main component of green propolis, was also detected. The geopropolis extracts showed good cell viability inhibition and selectivity indices in comparison to cisplatin used as an HCC treatment option. The antioxidant capacity of the geopropolis extracts was high and correlated with the cytotoxic effect against the HCC cells. Investigations into the mechanisms show the ability of the extracts to induce apoptosis and suppress the clonogenic potential of these cell lines. We also observed an inhibition of spheroid formation, viability, and morphology alterations. This is the first time the effects of geopropolis are described in a panel of HCC cell lines.

## 1. Introduction

Despite being the sixth most diagnosed cancer worldwide, hepatocellular carcinoma (HCC) remains the third leading cause of cancer-related deaths globally [1]. HCC is the most common primary cancer affecting the liver, accounting for approximately 90% of cases [2]. In general, HCC develops from a previous chronic liver disease, such as hepatitis B or C virus infection, exposure to aflatoxin B1, non-alcoholic fatty liver disease (NAFLD), and alcoholic cirrhosis [3].

Different molecular mechanisms involved in HCC, influenced by sites of carcinogenesis and the patient’s medical history, render each tumor nearly unique and present significant challenges for treatment. Treatment protocols for HCC are evaluated according to the stage of the tumor and the functionality of the organ and may involve one or more options, such as surgical resection of the tumor, the treatment option used when the liver functions remain unchanged; organ transplant, indicated when there is associated cirrhosis; ablation, a thermotherapy induced by microwaves, or heat, which directly affects the lesions and is recommended for patients not eligible for transplants; transarterial chemoembolization and transarterial radioembolization, which consist of the injection of chemotherapy drugs or microspheres of radioactive compounds directly into the tumor, aiming to cause ischemia and necrosis; and systemic treatments, such as sorafenib and cisplatin [4,5].

Brazilian geopropolis, derived from various species of stingless bees (meliponines), has attracted significant attention due to its complex composition and diverse pharmacological properties. The most commonly identified chemical classes in geopropolis include terpenoids and phenolic compounds, particularly flavonoids. However, less common chemical classes have also been reported, such as polyprenylated benzophenones, alkaloids, and phenylpropanoids [6]. This molecular diversity makes geopropolis a promising source of bioactive compounds. In fact, studies have highlighted the biological activities of geopropolis from different *Melipona* species, including antioxidant [7], anti-inflammatory [8], antimicrobial [8,9], and anticancer [10,11] properties.

In this study, we demonstrate that geopropolis extracts from the meliponine bees *Melipona mondury*, *Melipona marginata*, and *Melipona bicolor* show antitumor activity in HCC cells and notable selectivity for tumor cells. Mechanistically, they are able to induce apoptosis and suppress the clonogenic potential of these cell lines. In a 3D-cell culture model, the geopropolis extracts were either able to prevent the formation of spheroids or to alter the size, morphology and viability of formed ones. A phytochemical analysis, through UPLC-QTOF HRMS/MS revealed the predominance of flavonoids and diterpenes in the three samples. Interestingly, the phenylpropanoid artepillin C, a main component of green propolis, was also detected. Taken together, our results suggest that geopropolis could be a promising candidate for chemotherapy against HCC.

## 2. Materials and Methods

### 2.1. Geopropolis Extracts Preparation

The geopropolis produced by *M. mondury*, *M. bicolor*, and *M. marginata* were collected in January 2021 (Guapimirim, RJ, Brazil). The resinous material (200 g) was subjected to static maceration in 95% ethanol for 24 h at room temperature, in the absence of light. Subsequently, the solid phase of the samples was removed by filtration, while the liquid phase was stored for 24 h at −20 °C for wax precipitation. Finally, the liquid phase was filtered and subjected to drying in a rotary evaporator to obtain the following extracts: GEMB (geopropolis from *M. bicolor*), GEMG (geopropolis from *M. marginata*), and GEMM (geopropolis from *M. mondury*). For the antioxidant capacity assays, the extracts were prepared as stock solutions standardized at 100 mg/mL in DMSO. Working solutions were properly prepared in ultrapure water at a final concentration of 1 mg/mL. The geopropolis samples were duly registered in the National System for the Management of Genetic Heritage and Associated Traditional Knowledge (SISGEN, no. A236234).

### 2.2. Chemical Profiling of Geopropolis Extracts

The geopropolis extracts were analyzed using ultra-high-performance liquid chromatography coupled with high-resolution mass spectrometry (UPLC-QTOF HRMS/MS). Each sample was diluted in methanol and ultrapure water (8:2) to a concentration of 2.0 mg/mL, centrifuged, and then transferred to HPLC vials. Chromatographic separation was performed using a Shimadzu C-18 column (150 × 20 mm, 2.2 µm particles) on a Shimadzu Nexera 30 UPLC system. The mobile phase consisted of 0.1% formic acid in water (phase A) and acetonitrile (phase B), with a gradient from 3% to 80% phase B over 42 min at a flow rate of 0.4 mL/min. The QTOF Compact Bruker mass spectrometer was set up for electrospray ionization (ESI) in both negative and positive modes, covering a mass range of 100 to 1000 Daltons. Sodium formate was used as the calibrant. All solvents used were HPLC grade (Tedia Brazil, Rio de Janeiro, RJ, Brazil). The compounds were tentatively annotated using the equipment database and literature.

### 2.3. Antioxidant Capacity

The antioxidant capacity of the geopropolis extracts was evaluated using the ABTS^•+^ scavenging assay. The radical was generated with an ABTS (2,2’-azino-bis(3-ethylbenzothiazoline-6-sulfonic acid) working solution (0.0066 g K_2_S_2_O_8_ and 0.0384 g ABTS in 10 mL ultrapure water), prepared 16 h prior to the experimental procedure as previously described [12]. The extracts were dissolved at a concentration of 1 mg/mL in ultrapure water, and 10 μL aliquots were transferred in triplicate to 96-well microplates. Then, 190 μL of the ABTS^•+^ solution was added to each well. The absorbances were measured using a SpectraMax iD3 microplate reader (Molecular Devices, San Jose, CA, USA) at 720 nm. The results were expressed as a percentage of radical scavenging activity using the following formula: [(ABS_A_ − ABS_B_)/ABS_A_] × 100 where ABS_A_ is the absorbance of the ABTS^•+^ solution with water and ABS_B_ is the absorbance of the sample.

The ferric reducing antioxidant power (FRAP) of the samples was determined by adding 180 µL of the FRAP reagent [2 mL of 10 mM TPTZ solution in 6N HCl, 2 mL of 20 mM FeCl_3_ solution, and 20 mL of 300 mM acetate buffer, pH 3.6, and warmed to 37 °C] to 20 µL of the samples in a 96-well microplate. A calibration curve was constructed using different concentrations (15.1–500 μM) of FeSO_4_. The absorbance was measured at 595 nm using a Multiskan FC microplate reader (Thermo Scientific Inc., Waltham, MA, USA). The results were expressed in µmol Fe^2+^ per g [13].

### 2.4. Cell Culture

Hepatocellular carcinoma cells (Hep3B, HepG2, Huh-7, and PLC/PRF/5) were cultured in Eagle’s Minimum Essential Medium (EMEM) with 1% L-glutamine (Gibco Scientific, Grand Island, NY, USA). Hepatocytes isolated from the normal liver (AML-12) were cultured in a 1:1 mixture of Dulbecco’s Modified Eagle Medium (DMEM) and Ham’s F-12 medium (Sigma-Aldrich, San Louis, MO, USA) with the insulin-transferrin-sodium selenite media supplement (Sigma-Aldrich, San Louis, MO, USA). Human monocytic leukemia cells (THP-1) were differentiated into macrophages by incubation with 40 ng/mL of phorbol 12-myristiate-12 acetate (PMA, Sigma-Aldrich, San Louis, MO, USA) in RPMI-1640 medium with 10% fetal bovine serum and 0.1% gentamicin (10 mg/mL) for 48 h at 37 °C in 5% CO_2_. The medium of all cells was supplemented with 10% fetal bovine serum and 0.1% gentamicin (10 mg/mL), maintained at 37 °C in a humidified atmosphere containing 5% CO_2_. For all experiments, ethanol concentration (used to dilute geopropolis extracts) was normalized to 0.05%, including controls. Cells were purchased from the Rio de Janeiro Cell Bank (BCRJ, Rio de Janeiro, Brazil).

### 2.5. Cell Viability

Cells were transferred to 96-well microplates to 80% of confluency. Then, the cells were treated with different concentrations of the geopropolis extracts or cisplatin (solubilized in PBS), in serial dilution. For HCC cell treatment, the concentrations of GEMM and GEMQ were 0.78 to 25 µg/mL, the concentrations of GEMG and GEMB were 3.9 to 31.3 µg/mL, and those of cisplatin were 0.39 to 25 µg/mL. The serial dilution for THP-1 and AML-12 was from 1.56 to 100 µg/mL with GEMM, GEMG and GEMB, and GEMQ and cisplatin. After 48 h, an MTT solution (0.5 mg/mL, Sigma-Aldrich, San Louis, MO, USA) was added to the microplate wells, which were incubated for 2–4 h. Formazan crystals were diluted with DMSO and the plates were analyzed with SpectraMax Paradigm multimode microplate reader (Molecular Devices, San Jose, CA, USA) at 570 and 650 nm [14]. The concentration required to inhibit 50% of cell viability (IC_50_) was calculated using the Prism 8.0 program (GraphPad Software, Boston, MA, USA), and the selectivity index (SI), which estimates the relative toxicity of the extracts, was calculated as the ratio between the IC_50_ values of the non-tumor cell line and the tumor cell lines [15].

### 2.6. Colony Formation Assay

In a 6-well plate, Hep3B and Huh-7 cells (1000 cells/well) were treated with different concentrations (in Hep3B and Huh-7 cells, respectively) of GEMB (5 and 17 µg/mL), GEMG (9 and 16 µg/mL), and GEMM (6 and 7 µg/mL). The plates were incubated at 37 °C and 5% CO_2_ until colonies formed. The colonies were fixed and stained with crystal violet solution (0.5% crystal violet and 25% methanol in water).

### 2.7. Annexin V-FITC/ Propidium Iodide Apoptosis

Hep3B and Huh-7 cells were plated in 24-well plates to a confluence of 60%. The cells were treated with different concentrations of GEMB (3 and 8 µg/mL), GEMG (5 and 8 µg/mL), and GEMM (3 and 4 µg/mL) in Hep3B and Huh-7 cells, respectively. After 24 h, the apoptosis assay was performed according to the instructions of the Apoptosis Detection Kit (ThermoFisher, Waltham, MA, USA), using annexin V-FITC and propidium iodide in a Countess II FL cell counter (ThermoFisher, Waltham, MA, USA).

### 2.8. 3D-Cell Culture

Fifty microliters of 1% agarose (ref #A9414, suitable for cell culture, Sigma-Aldrich, San Louis, MO, USA) were added to 96-well plates and, after solidifying, 4 × 10^3^ Hep3B and Huh-7 cells per well were added to each plate. For the assay in which cells were treated immediately after plating, GEMM, GEMG, and GEMB at 5 or 15 µg/mL were added to the wells. The plates were centrifuged at 400× *g* for 10 min and incubated for 72 h at 37 °C in 5% CO_2_. For the assay in which spheroids were previously prepared and then treated, after adding the cells to the wells, the plate was centrifuged at 400× *g* for 10 min and incubated for 72 h at 37 °C in 5% CO_2_. Then, the spheroids were treated with 5 and 15 µg/mL of GEMM, GEMG, and GEMB for 48 h. At the conclusion of both assays, images were obtained with an EVOS brightfield microscope (EVOS M5000 Cell Imaging System, Life Technologies, Waltham, MA, USA) [16], and viability was measured through the acid phosphatase assay (APH). After a 2 h incubation with 100 uL of APH (containing 2 mg/mL of p-nitrophenyl phosphate ( Sigma-Aldrich, San Louis, MO, USA) and 0.1% Triton X in 0.1 M citric acid), 10 μL of NaOH (1 M) was added, and it was analyzed at 405 and 630 nm [17].

### 2.9. Statistical Analyses, IC_50_, Correlation Analyses and Selectivity Index Calculation

Statistical analyses were performed using SigmaPlot for Windows version 12 (Systat Software, Inc, San Jose, CA, USA). The results were expressed as means and standard error of triplicate determinations and analyzed by Student’s t-test or ANOVA and Tukey, as described in figures legends. The IC_50_ values were calculated using GraphPad Prism v. 8.0.1. The correlation analyses were performed using the Past 4.03 software (University of Oslo, Oslo, NO, USA). A *p*-value < 0.05 was considered significant. The selectivity index was estimated as the ratio between the IC_50_ value of non-cancerous cells and the IC_50_ value of cancerous cells [15], for each geopropolis extract.

## 3. Results and Discussion

### 3.1. Chemical Profile of Geopropolis Extracts

Through UPLC-QTOF HRMS/MS analysis, 14 different substances were annotated, predominantly flavonoids (*n* = 7) and diterpenes (*n* = 4), along with two phenylpropanoids and one lignan. These compounds were present in varying amounts across the extracts, with their relative percentages determined based on the major peak (100%) of a given compound compared to others, as shown in Table 1. In the GEMB extract, the major component was annotated as cupressic acid (100% peak intensity), followed by ferreirin (92.6%) and 15-acetoxyisocupressic acid (84.7%).

Other substances included artepillin C (15%), isocupressic acid (14.4%), and pinoresinol (10.5%). In the GEMG extract, the major compound was 15-acetoxyisocupressic acid (100% peak intensity), followed by cupressic acid (82.1%) and dihydrokaempferide (71.9%). Additional substances were identified, including ferreirin (70.9%), artepillin C (15.8%), and isocupressic acid (0.3%). For the GEMM extract, the main constituent was dihydrokaempferide (100% peak intensity), followed by 15-acetoxyisocupressic acid (97.1%) and ferreirin (91.7%). The presence of cupressic acid (87.8%), isocupressic acid (21.3%), and artepillin C (10.7%) was also observed. Interestingly, dihydrokaempferide as presentw in a lower proportion in GEMG (7.7%).

The presence of compounds from phenolic and terpenoid chemical classes has been corroborated by previous studies on Meliponini geopropolis, as reviewed by Lavinas et al. [6]. However, variations in the chemical profiles of geopropolis are frequently reported. For instance, *M. mondury* geopropolis samples from two different areas in southern Brazil were found to contain 4-aminobenzoic acid, protocatechuic acid, and vanillin as the main phenolic components, with one sample showing notably high levels of syringaldehyde. Interestingly, some of these compounds were identified for the first time as constituents of Meliponini geopropolis [18]. In the present study, ferreirin was the main phenolic component in the extracts, followed by artepillin C. Ferreirin is an isoflavonoid already detected in some plant species, including *Gynerium sagittatum* (Poaceae) [19] and the legume species *Swartizia polyphylla* [20,21] and *Cajanus cajan* [22]. However, to the best of our knowledge, this is the first report of its presence in the stingless bee geopropolis. In turn, artepillin C is uncommon in other types of propolis as it is primarily found in Brazilian green propolis produced by *A. mellifera* bees from the resin of *Baccharis dracunculifolia* [23]. Nonetheless, Surek et al. [24] identified this diprenylated phenylpropanoid in ethanol extract of *Scaptotrigona bipunctata* propolis. Here, artepillin C was annotated in the geopropolis extracts of three different stingless bee species. These findings highlight the potential of stingless bee geopropolis as a source of artepillin C, which is widely recognized for its diverse pharmacological activity [23].

The main diterpenoids identified in this study have also been reported in Brazilian propolis. Cupressic acid, isocupressic acid, and 15-acetoxyisocupressic acid have been successfully isolated from brown propolis, along with other terpenoids [25,26]. In Meliponini propolis, cupressic acid and isocupressic acid were previously detected in extracts from *Melipona quadrifasciata* and *Plebeia remota* [23]. Up to this moment, 15-acetoxyisocupressic acid has not been identified in Meliponini propolis or geopropolis.

### 3.2. Antioxidant Capacity of Geopropolis Extracts

**Figure 1:**
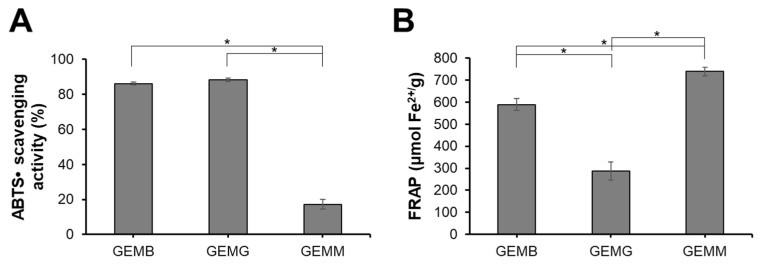
Antioxidant capacity of GEMB, GEMG, and GEMM. A working solution of each extract was standardized to 1 mg/mL. (**A**) ABTS^•+^ scavenging activity of the geopropolis samples. (**B**) Ferric reducing power of the geopropolis samples. Asterisks indicate statistical differences between samples, where * *p* < 0.05 (ANOVA and Tukey).

The antioxidant capacity of geopropolis extracts was also evaluated (Figure 1). GEMB and GEMG exhibited the highest ABTS^•+^ scavenging activities, with values of 86 ± 0.8% and 88 ± 1.1%, respectively, while GEMM showed lower activity at 17.3 ± 2.7%. For the FRAP assay, GEMM demonstrated the highest activity with 738 ± 18 µmol Fe^2+^/g, followed by GEMB at 589 ± 27 µmol Fe^2+^/g and GEMG at 287 ± 41%. It is noteworthy that the antioxidant capacity of the extracts can vary depending on the evaluation method, a phenomenon commonly observed in such studies [27]. For instance, while GEMM exhibited lower radical scavenging potential, it demonstrated a strong ability to reduce prooxidant metals. This highlights the importance of assessing antioxidant capacity using multiple methodologies to capture a more comprehensive understanding of the extracts’ antioxidant properties. These findings align with the chemical profile described above, highlighting the role of predominant phenolic compounds. Geopropolis is widely recognized for its potent antioxidant capacity, as evidenced by various assays, including ABTS and FRAP. The hydroalcoholic extract of *M. mondury* geopropolis and its fractions has previously been shown to possess antioxidant capacity, demonstrating radical scavenging activity at low concentrations comparable to standard antioxidants such as Trolox and gallic acid [28]. In addition, geopropolis extracts from *M. bicolor* and *M. marginata* demonstrated promising antioxidant capacity through various methods, including the FRAP assay, in which values ranged from 1.25 to 3.23 µg Fe^2+^/g and 0.01 to 3.17 µg Fe^2+^/g, respectively. The effectiveness of geopropolis as an antioxidant agent is closely tied to its composition, which can vary significantly. This variation is influenced by multiple factors, including the botanical source and the extraction method used, highlighting the importance of these variables in determining the antioxidant potential to of geopropolis extracts [29].

### 3.3. Cytotoxic Effect of Geopropolis Extracts on HCC Cell Lines in the 2D-Cell Culture Model

**Figure 2:**
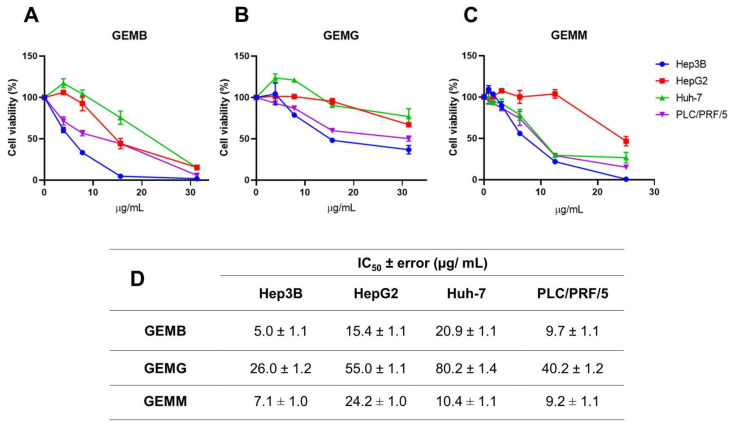
Geopropolis extracts are cytotoxic to HCC cell lines. Hep3B, HepG2, Huh-7, and PLC/PRF/5 cell lines were treated with increasing concentrations of GEMB (**A**), GEMG (**B**), and GEMM (**C**) for 48 h and cell viability was analyzed by MTT. (**D**) The IC_50_ values were calculated and are listed in (**D**).

The cytotoxic activities of geopropolis extracts were investigated in hepatocellular carcinoma (HCC) cell lines through the cellular metabolic activity measured by the MTT assay. Through concentration–response curves, we observed that GEMB (Figure 2A), GEMG (Figure 2B), and GEMM (Figure 2C) were cytotoxic to Hep3B (blue), HepG2 (red), Huh-7 (green), and PLC/PRF/5 (purple), reducing the cell viability of the cell lines after 48 h of treatment. These curves allowed for the determination of the half-maximal inhibitory concentration (IC_50_) of the extracts, listed in Figure 2D. In general, the Hep3B cell line showed the greatest reduction in viability with lower concentrations of the extracts, especially GEMM (7.0 ± 1.0 µg/mL) and GEMB (5.0 ± 1.1 µg/mL). GEMG was the extract that obtained the highest IC_50_ values of 26.0 ± 1.2; 55.0 ± 1.1; 80.0 ± 1.4 and 40.2 ± 1.2 µg/mL, for the Hep3B, HepG2, Huh-7, and PLC/PRF/5 cell lines, respectively.

**Figure 3:**
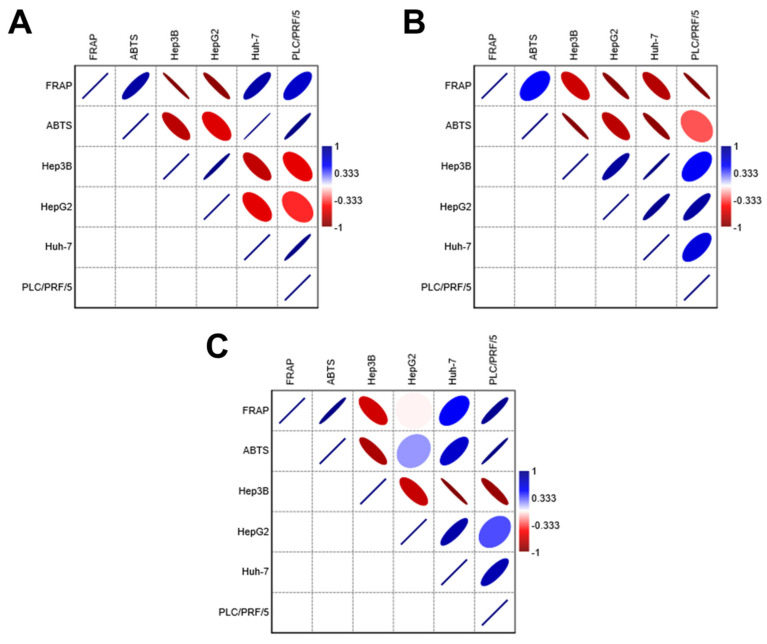
Correlation analyses of the antioxidant capacity and cytotoxic effects of geopropolis extracts. Both FRAP and ABTS data were subjected to correlation analysis with the IC_50_ values of each HCC cell line. (**A**) GEMB, (**B**) GEMG, and (**C**) GEMM. Correlation coefficients range from 1 (strong positive correlation, blue) to −1 (strong negative correlation, red). The color intensity reflects the strength of the correlation: darker shades of blue or red indicate stronger correlations, while lighter shades or neutral colors indicate weaker correlations. The correlation matrices for GEMB, GEMG, and GEMM are in the Supplementary Material (Tables S1–S3, respectively).

The antioxidant data from the geopropolis extracts were subjected to correlation analyses to gain new insights into their biological activity against HCC cell lines (Figure 3 and Tables S1–S3). The results show that GEMG antioxidant capacity displays a strong negative correlation with the cytotoxic concentrations required to reach 50% of cell viability for all HCC cell lines (intense red color, values < −0.333), regardless of the antioxidant assay used. This finding aligns with the following expectations: the higher the antioxidant potential, the more active the extract, as indicated by lower IC_50_ values. Therefore, a high antioxidant potential should correspond to lower effective concentrations of the extracts. The ferric reducing power and ABTS^•+^ scavenging activity of GEMB showed negative correlations with Hep3B and HepG2 cell lines but not with Huh-7 and PLC/PRF/5 lines. In turn, the antioxidant capacity of GEMM negatively correlated only with Hep3B. Strong positive correlations (intense blue color, values > 0.333) were also observed, suggesting that the antioxidant capacities of GEMB and GEMM may differently affect various HCC cell types. Notably, the antioxidant capacity of natural products may not always reflect their actual bioactivity, including their cytotoxic effectiveness, which could partially explain the divergences observed in the correlation analyses. However, antioxidant capacity assays remain valuable tools for providing initial evidence of the antioxidant potential of new drug candidates, including those of natural origin. Additionally, the antioxidant capacity of bioactive candidates should be assessed carefully, using multiple antioxidant-based assays to gain a more comprehensive understanding of their biological value.

### 3.4. Cytotoxic Effects of Geopropolis Extracts on Non-Cancerous Cell Lines and Selectivity Indices

**Figure 4:**
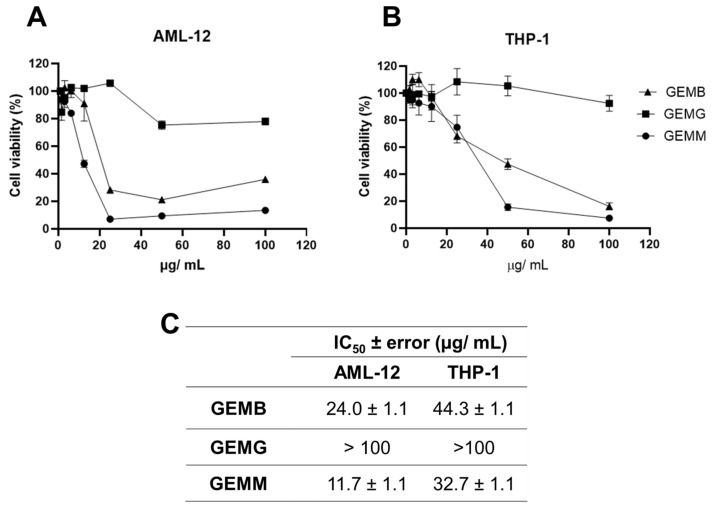
Cytotoxicity of geopropolis extracts in human non-cancerous cell lines. The concentration–response curves were performed with non-tumor cells (**A**) AML-12 and (**B**) THP-1 were treated with GEMB, GEMG, and GEMM. After 48 h, an MTT cell viability assay was performed. (**C**) IC_50_ values were obtained from the curves. *n* = 3.

Since a correlation between cytotoxicity to cancerous cells and the antioxidant capacity of some of the geopropolis extracts was established, we moved further on the investigation of their anticancer potential. We started by analyzing another set of cell viability assays in two non-cancerous cell lines. Our objective was to compare the effects of the extracts between the cancerous and non-cancerous cell lines and estimate their selectivity. The concentration–response curves obtained for AML-12 mouse hepatocytes (Figure 4A) and THP-1 human macrophages (Figure 4B) demonstrated a reduction in viability at some point, except for GEMG, which showed no significant reduction in cell viability in all the concentrations tested. For this reason, the IC_50_ values were not calculated. The IC_50_ ± error values observed for the geopropolis extracts were 24.0 ± 1.1 for AML-12, 44.3 ± 1.1 µg/mL for THP-1 with GEMB, and 11.7 ± 1.1 for AML-12, and 32.7 ± 1.1 µg/mL for THP-1 with GEMM (Figure 4C).

**Figure 5:**
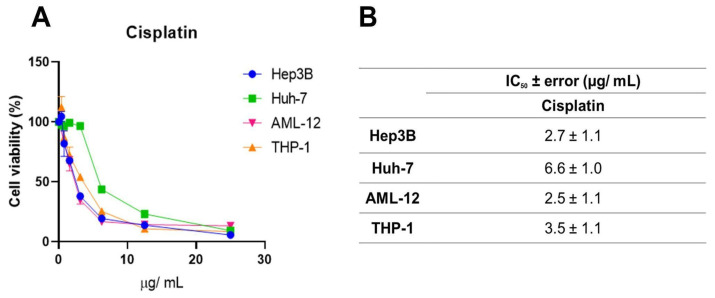
Cell viability inhibition by cisplatin in HCC and non-cancerous cell lines. (**A**) Hep3B, Huh-7, AML-12, and THP-1 were treated with increasing concentrations of cisplatin. After 48 h, cytotoxicity was measured through the MTT assay, and the IC_50_ values (*n* = 3) were calculated (**B**).

Systemic chemotherapeutics, such as cisplatin, occupy a prominent place in the treatment of several malignant neoplasms due to their effectiveness, but they trigger serious adverse effects [30]. This, along with the need to increase the rates of success for cancer treatments, brings forth the search for new anticancer drug candidates. Here, we used cisplatin as a parameter to evaluate the efficacy of the geopropolis extracts. For this, one more set of MTT assays (Figure 5A) was performed with cisplatin using Hep3b and Huh-7 HCC cell lines and the two non-cancerous cell lines with increasing concentrations of the three extracts, for 48 h. For this, we chose to use only two of the cancerous cell lines, Hep3B, which presented the best cytotoxic effects and a correlation with antioxidant capacity (Figure 2 and Figure 3) and was also a Hepatitis B genome carrier, and Huh-7, a cell line free from viral genome integration that showed a different cytotoxic profile. A reduction in the viability of Hep3B (blue), Huh-7 (green), AML-12 (pink), and THP-1 (orange) after 48 h was observed. IC_50_ values (Figure 5B) of 2.7 ± 1.1; 6.6 ± 1.0; 2.5 ± 1.1 and 3.5 ± 1.1 µg/mL were obtained for Hep3B, Huh-7, AML-12, and THP-1 cell lines, respectively. For the HCC cell lines, the IC_50_ values were slightly higher than those observed for Hep3b for GEMB and GEMM, but in the same order of magnitude. On the other hand, for the non-cancerous cell lines, similarly low values were obtained, which indicated low selectivity for the cancerous cells. This lack of selectivity shown by cisplatin was related to organ-specific toxicity and, also, to its side effects. For this reason, we estimated the selectivity indices for the geopropolis extracts in comparison to cisplatin (Table 2).

The SI values (Table 2) are determined by calculating the ratio of IC_50_ values between non-cancerous (here, AML-12, a hepatocyte cell line, and THP-1, a monocyte cell line) and the previously used cancerous cell lines (Hep3B and Huh-7). An SI greater than 1 indicates that the compound is more toxic to cancerous cells than to non-cancerous cells [15]. The highest SI value was 8.86 ± 0.24 (GEMB), for the THP-1/Hep3B ratio. Overall, GEMB showed the best selectivity index for Hep3B and Huh-7, with values almost four times higher than the SI of cisplatin. As explained before, the IC_50_ value of GEMG was not possible to estimate, thus, the SI of GEMG was not detected (n.d.). In general, all geopropolis extracts demonstrated a good SI when compared to cisplatin, showing greater selectivity toward tumor cells. Interestingly, the values observed in this analysis confirm what the comparison between IC_50_ values showed us: the geopropolis extracts are 4 to 6 times more selective than cisplatin to HCC cells.

### 3.5. Effects of Geopropolis Extracts on a 3D-Cell Culture Model

Three-dimensional cell culture models are used in an attempt to resemble tumor conditions, compared to 2D cultures. They allow for the observation of the interactions between cells and with the extracellular matrix, generating a gradient of both nutrients and oxygenation, resulting in a physiology that is closer to the in vivo context, even though other tumor microenvironment components are not present. This could aid the comprehension of the pharmacological response, improving the analysis of the efficacy and toxicity of compounds [31].

**Figure 6:**
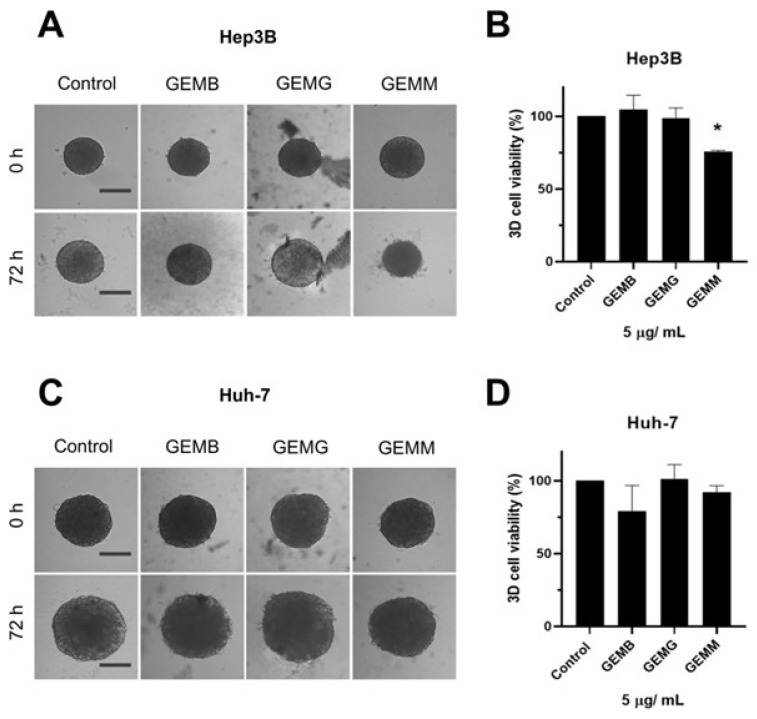
Geopropolis extracts alter the structure of Hep3B and Huh-7 spheroids. Previously formed Hep3B (**A**) and Huh-7 (**C**) spheroids were treated with GEMM, GEMG, and GEMB at 5 and 15 µg/mL. After 72 h, spheroids were assessed for viability using the acid phosphatase method (Hep3B, (**B**); Huh-7, (**D**)). Scale bar, 100 µm. Representative images are shown (*n* = 4), * *p* < 0.05 (Student’s *t*-test).

Two types of protocols for analyzing the effects of geopropolis extracts on the 3D-cell culture were adopted. The first one was performed to observe the effects of geopropolis extracts on spheroid morphology and cell viability. For this, Hep3B (Figure 6A) and Huh-7 (Figure 6B) spheroids were prepared and cultivated for 72 h before being treated with GEMB, GEMG, and GEMM at 5 μg/mL. Then, after another 72 h, Hep3B and Huh-7 spheroids were scanned for morphological alterations.

We observe that Hep3B spheroids treated with GEMB and GEMM presented less growth and seemed smaller than their 0 h treatment counterparts. Especially for GEMM, a size reduction is observed, and the outer layer of the spheroid, which is responsible for cell proliferation due to the higher contact with oxygen and nutrients, seems to be more damaged than in the control spheroids. For Huh-7 spheroids, size reduction is not so prominent although an apparent reduction is observed for GEMB and GEMM in comparison to the control and GEMG. For the tree extracts, spheroids also show a larger necrotic core the darker central core, which lacks nutrients and oxygen [32,33], except for GEMG in Hep3B cells, which show a visible intermediate area, with a smaller necrotic core than the control. Also, the viability of the cells in the spheroids was measured. In all conditions tested, the only one that produced a statistically significant reduction in cell viability was Hep3B treated with GEMM.

**Figure 7:**
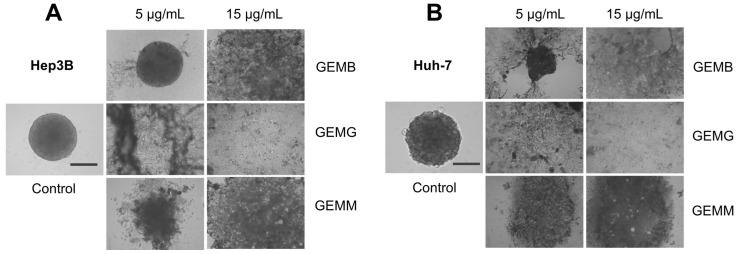
Geopropolis extracts prevent the formation of HCC spheroids. Hep3B (**A**) and Huh-7 (**B**) spheroids were treated with GEMB, GEMG, and GEMM (5 and 15 μg/mL). After 72 h, images were analyzed and, except for GEMB at 5 μg/mL, geopropolis extracts prevented the formation of HCC spheroids. Scale bar, 100 µm. Representative images of three experiments are shown.

Since already-formed spheroids treated with geopropolis extracts presented only moderate changes when treated, we evaluated their effects on other assays that correlate with a tumo development. We analyzed the effects of the geopropolis extracts on the assembly of spheroids. For this, in the first 72 h after being plated, cells were incubated with GEMB, GEMG, and GEMM at 5 and 15 μg/mL and the formation of spheroids was observed. The results show that GEMB failed to inhibit spheroid formation for both cell lines at 5 μg/mL, but had positive results at 15 μg/mL. On the other hand, the two concentrations of GEMG and GEMM tested successfully inhibited the formation of Hep3B (Figure 7A) and Huh-7 (Figure 7B) spheroids compared to their respective controls.

### 3.6. Geopropolis Extracts Impair the Formation of HCC Cell Colonies

**Figure 8:**
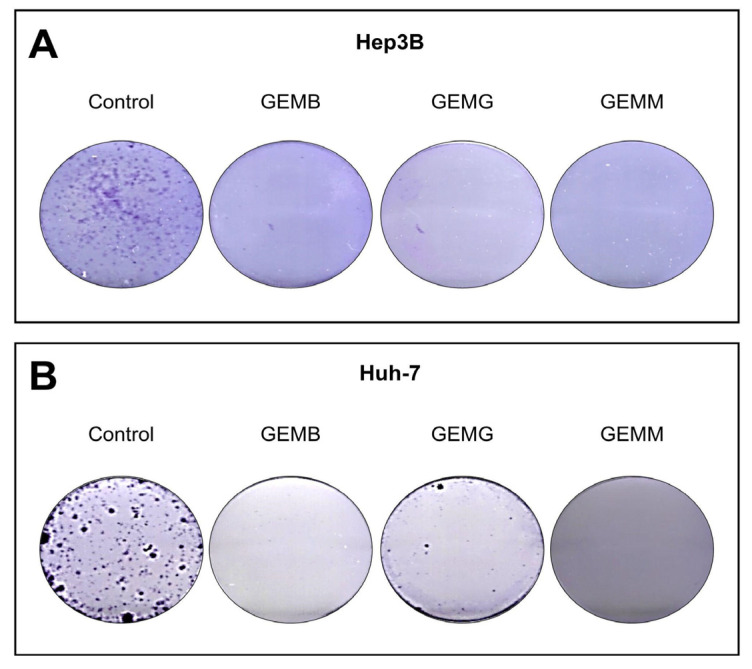
Geopropolis suppresses the formation of colonies of HCC cells. Hep3B (**A**) and Huh-7 (**B**) cells treated with GEMB ((**A**), 5 and (**B**), 17 μg/mL), and, GEMG ((**A**), 9 and (**B**), 16 μg/mL), and GEMM ((**A**), 6 and (**B**), 7 μg/mL) show a complete suppression of colony formation in the concentrations tested. Representative images of three experiments are shown.

The clonogenic assay involves the ability of a single cell to adhere to a surface and form a colony with more than 50 cells. Thus, the effectiveness of the treatment can be observed by the loss of this ability, which is related to the reduction of chances of metastases [34]. Not all cells present in a tumor are clonogenic; however, the ability to eradicate this type of cell is an important feature for a drug candidate [35]. Cells were counted using Trypan blue and a total of 1000 viable cells were plated and then treated with concentrations related to the IC_50_ values of GEMB and GEMM, which were the lowest. We observed that the three geopropolis extracts were able to completely abolish colony formation in both Hep3B (Figure 8A) and Huh-7 cells (Figure 8B), compared to their respective controls.

### 3.7. Apoptosis Induction by Geopropolis Extracts

**Figure 9:**
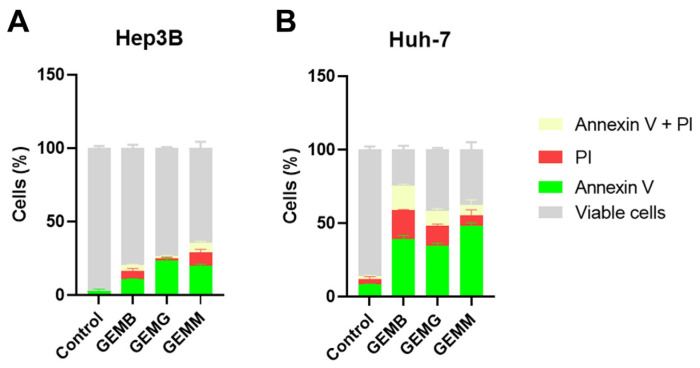
Geopropolis extracts induce apoptosis in HCC cell lines. Apoptosis was measured using the annexin V-FITC and propidium iodide method. The amount of cells stained with annexin V is shown in green. Cells stained with propidium iodide (PI) are represented in red. Cells with both stainings are represented in pale yellow and grey represents the cells that were not stained and remained viable. Hep3B cells (**A**) and Huh-7 (**B**) cells were treated with GEMB (3 and 8 µg/mL), GEMG (5 and 8 µg/mL), and GEMM (3 and 4 µg/mL), respectively. *n* = 3.

The annexin V/PI assay identifies early events of apoptosis, through the presence of cells to which annexin V binds to phosphatidylserine exposed on the cell surface, while propidium iodide (PI) stains necrotic or late apoptotic cells since it only penetrates cells with damaged membranes [36]. Figure 9A,B shows the induction of apoptosis in Hep3B and Huh-7 cells, respectively. The concentrations used corresponded to half of the IC_50_ values, and cells were treated for 24 h, instead of the 48 h from the cytotoxicity assays, with the aim to visualize the initial process of apoptosis. Overall, we observe that all geopropolis extracts induce apoptosis in the HCC cell lines compared to the control. However, the effects observed for Huh-7 cells for the three geopropolis extracts were more prominent than those observed for Hep3B cells with the concentrations and time selected for this experiment.

Dos Santos et al. described antioxidant and antiproliferative effects for a *M. mondury* hydroethanolic extract on different cancer cell lines, including HepG2, one of the HCC cell lines used here [28]. Geopropolis that originated from *Melipona fasciculata* has been described to promote cytotoxicity, proapoptotic, antioxidant, and anti-inflammatory effects against lung and ovarian cancer [37] and, also, against canine osteosarcoma in vitro [38]. The anticancer and antiproliferative effects of geopropolis produced by other stingless bee species were also described, alone [39,40] or in combination with cytotoxic drugs [11].

Among the compounds annotated in the geopropolis extracts of the three different stingless bee species used here, artepillin C, the main component of Brazilian green propolis, was described to act through several different mechanisms to exhibit its anticancer properties: apoptosis induction, cell cycle arrest, and the inhibition of p21-activated kinase 1 (PAK1) are the main described ones [41,42]. The other phenylpropanoid detected, caffeic acid phenylethyl ether, had its radical scavenging and anticancer activities reported [43,44]. Pinoresinol [45] was shown to induce apoptosis and inhibit invasion in HepG2 cells and to exhibit a pro-oxidant activity that led to antitumoral effect on breast cancer cells [46]. The main diterpenoid compounds in our extracts, cupressic acid, isocupressic, acid and 15-acetoxyisocupressic acid, to the best of our knowledge, have not had their effects described on cancer in the isolated form but were detected in propolis from different species with cytotoxic activity [24].

Flavonoids such as betuletol, kaempferide, isorhamnetin, and ferreirin, detected in our geopropolis samples (Table 1), have been reported to contribute greatly to the antioxidant and anticancer activities of propolis [29,47,48,49]. Dihydrokaempferide, the main compound annotated in GEMM, which is in lower proportion in GEMB, was first isolated from *Chromolaena odorata*, and its cytotoxic effects were tested on various cancer cell lines. It showed limited cytotoxicity compared to other flavonoids such as kaempferide [50]. Of note, quercetin dimethyl ether was not detected in GEMG. Quercetin and its derivatives have chemopreventive [51] and anticancer properties [52]. Also, quercetin is a p-glycoprotein inhibitor that enhances doxorubicin effects on breast cancer cells [53] and the combination of kaempferol with quercetin was described to increase their anticancer properties [54]. Thus, it is possible that the combination of specific components of the geopropolis extracts could lead to a synergistic effect that makes them have different effects in this panel of cell lines. However, it is important to notice that although these compounds share a high structural resemblance, their mechanisms of action, targets, and bioavailability may vary according to the metabolites produced and substitutions found [55]. It is also important to consider that geopropolis properties could overcome the effects of their isolated components by acting on diverse mechanisms simultaneously, as an active pharmaceutical ingredient with possible synergisms [56]. The combination of a great number of antioxidants with different mechanisms of action could also promote a pro-oxidant effect on tumor cells, leading to increased cytotoxicity [57,58]. It is known that flavonoids show antioxidant activity in non-tumor cells while exhibiting pro-oxidant effects by increasing oxidative stress levels in cancer cells. This dual activity results in the inhibition of cell proliferation signaling pathways, suppression of pro-inflammatory cytokines, and facilitation of apoptotic and necrotic processes, as well as the activation of autophagy [59].

## 4. Conclusions

In this work, we describe the potential anticancer properties of geopropolis extracts from different species of stingless bees from Brazil in HCC cell lines. GEMB, GEMG, and GEMM demonstrated differential capacities to induce cytotoxicity to HCC cell lines in a 2D-cell culture model, with GEMB and GEMM presenting the lowest IC_50_ values in the Hep3B cell line. These effects correlate with the antioxidant effects produced by the extracts, especially for GEMB and GEMG in Hep3B and HepG2 cell lines and for Hep3B cells with GEMM. Interestingly, the best selectivity indices were observed with Huh-7 cells, but GEMM also showed a good IS on Hep3B cells. In spite of the lower SI values of GEMM in the other cell lines, it is important to notice that all geopropolis extracts exhibited greater SI values than cisplatin, a drug used in HCC chemotherapy. In the 3D-cell culture model used here, a more complex environment is produced in terms of drug and nutrient availability for cells. We observed that GEMM was the only extract able to significantly reduce cell viability of Hep3B spheroids, as well as their size and outer layer morphology, compared to the control. GEMM, as well as GEMG, was successful in inhibiting spheroid formation, which is in line with the ability of the geopropolis extracts to suppress colony formation and induce apoptosis in the cell lines tested. Our results indicate the anticancer potential of GEMM, either as an active pharmaceutical ingredient or a source for the isolation of specific compounds, for HCC and the high antioxidant capacity of geopropolis extracts, which could be related to their mechanism of action. However, further experiments to investigate specific targets and the combination, in vitro and in vivo, with currently used chemotherapeutic drugs to enhance their efficacy and reduce their side effects should be useful to determine their clinical potential for HCC.

## Figures and Tables

**Table 1:** The main compounds detected by UPLC–QTOF HRMS/MS of GEMB, GEMG, and GEMM.

Compound Class	Mass *m*/*z*	Mass Error (mDa)	Isotopic Fitting (mSigma)	RT (min)	Molecule	Molecular Formula	Relative Intensity * (%)		
							GEMB	GEMG	GEMM
Flavonoid	329.1391	0.9	19.7	12.2	Betuletol	C_17_H_14_O_7_	1.9	1.8	2.0
Flavonoid	299.0918	−0.6	4.1	12.7	Kaempferide	C_16_H_12_O_6_	1.5	1.3	1.8
Phenylpropanoid	283.0607	1.5	24.4	13.2	Caffeic acid Phenylethyl ether	C_17_H_16_O_4_	1.6	1.2	1.5
Lignan	357.1333	2.9	13.7	14.2	Pinoresinol	C_20_H_22_O_6_	10.5	7.9	9.9
Flavonoid	329.1396	1.5	16.9	15.5	Quercetin dimethyl ether	C_17_H_14_O_7_	3.2	ND	3.8
Flavonoid	255.0664	0.4	24.2	15.9	Pinocembrin	C_15_H_12_O_4_	2.7	3.1	1.7
Diterpenoid	333.2061	1.5	14.0	17.0	Agathic acid	C_20_H_30_O_4_	7.6	6.1	8.8
Diterpenoid	319.2273	−0.5	0.6	17.3	Isocupressic acid	C_20_H_32_O_3_	14.4	20.3	21.3
Diterpenoid	319.2276	0.6	5.7	17.9	Cupressic acid	C_20_H_32_O_3_	100	82.1	87.8
Flavonoid	315.1956	1.3	17.0	18.5	Isorhamnetin	C_16_H_12_O_7_	4.3	2.5	4.3
Phenylpropanoid	299.2018	−0.6	4.1	19.2	Artepillin C	C_19_H_24_O_3_	15	15.8	10.7
Diterpenoid	361.2383	0.3	1.6	19.8	15-Acetoxyisocupressic acid	C_22_H_34_O_4_	84.7	100	97.1
Flavonoid	301.2171	0.7	4.6	20.7	Dihydrokaempferide	C_16_H_14_O_6_	7.7	71.9	100
Flavonoid	301.2174	−0.2	3.3	21.1	Ferreirin	C_16_H_14_O_6_	92.6	70.2	91.7

ND: Not detected; * Abundance relative to the most intense identified *m*/*z*.

**Table 2:** Selectivity indices of the non-tumor cell lines, AML-12 and THP-1, in relation to the HCC cell lines, Hep3B and Huh-7.

	Selectivity Index AML-12		Selectivity Index THP-1	
	IC_50_ AML-12/ IC_50_ Hep3B	IC_50_ AML-12 /IC_50_ Huh-7	IC_50_ THP-1/ IC_50_ Hep3B	IC_50_ THP-1/ IC_50_ Huh-7
GEMB	4.80 ± 0.27	1.15 ± 0.1	8.86 ± 0.24	2.12 ± 0.08
GEMG	n.d.	n.d.	n.d.	n.d.
GEMM	1.65 ± 0.23	1.13 ± 0.2	4.61 ± 0.17	3.14 ± 0.14
Cisplatin	0.93 ± 0.85	0.38 ± 0.32	1.30 ± 0.72	0.53 ± 0.47

n.d. not determined due to IC_50_ > 100 μg/mL.

## Data Availability

The raw data necessary to support the results of this article will be promptly made available by the authors upon request.

## Supplementary Materials

The following supporting information can be downloaded at: https://www.mdpi.com/article/10.3390/antiox14020141/s1, Table S1: Correlation matrix between antioxidant capacity of GEMB and cytotoxicity against HCC cell lines.; Table S2: Correlation matrix between the antioxidant capacity of GEMG and cytotoxicity against HCC cell lines.; Table S3: Correlation matrix between the antioxidant capacity of GEMM and cytotoxicity against HCC cell lines.
