# Supplementary material for: Brazilian Stingless Bee Geopropolis Exhibit Antioxidant Properties and Anticancer Potential Against Hepatocellular Carcinoma Cells
Source: Antioxidants (Basel). 2025 Jan 24;14(2):141. doi: 10.3390/antiox14020141 (PMC11851454; doi:10.3390/antiox14020141)
Supplement: Supplementary file 1 [file antioxidants-14-00141-s001.zip › antioxidants-3441446-supplementary.pdf]

Article

Brazilian Stingless Bee Geopropolis Exhibit Antioxidant properties and Anticancer Potential Against Hepatocellular Carcinoma Cells

Mariana Muniz da Paz[1], Kamila Marques Sette[1], Raissa Eduardo dos Santos[1], Ana Luiza Barbosa e Vasconcelos[1], Danielly Ferraz da Costa[2], Ana Claudia F. Amaral[3], Igor Almeida Rodrigues[4]* and Luciana Pereira Rangel[4]*

[1] Programa de Pós Graduação em Ciências Farmacêuticas, Faculdade de Farmácia, Universidade Federal do Rio de Janeiro, Rio de Janeiro, RJ, 21941-902, Brazil

[2] Instituto de Nutrição, Universidade do Estado do Rio de Janeiro, Rio de Janeiro, RJ, 20550-013 , Brazil.

[3] Farmanguinhos, Fundação Oswaldo Cruz, Rio de Janeiro, RJ, 21041-000, Brazil.

[4] Faculdade de Farmácia, Universidade Federal do Rio de Janeiro, Rio de Janeiro, RJ, 21941-902, Brazil

* Correspondence: igor@pharma.ufrj.br (I.A.R.), lprangel@pharma.ufrj.br (L.P.R.)

Supplementary Material

Table S1. Correlation matrix between antioxidant capacity of GEMB and cytotoxicity against HCC cell lines.

	FRAP	ABTS	Hep3B	HepG2	Huh-7	PLC/PRF/5
**FRAP**	1	0.85785	-0.98694	-0.93831	0.8391	0.7093
**ABTS**	0.85785	1	-0.76386	-0.62722	0.99937	0.97073
**Hep3B**	-0.98694	-0.76386	1	0.98176	-0.7405	-0.58648
**HepG2**	-0.93831	-0.62722	0.98176	1	-0.59922	-0.42178
**Huh-7**	0.8391	0.99937	-0.7405	-0.59922	1	0.97863
**PLC/PRF/5**	0.7093	0.97073	-0.58648	-0.42178	0.97863	1

The data are expressed as the correlation coefficient, r. An r value close to +1 or -1 indicates a strong relationship, while a value near 0 indicates a weak or no relationship.

Table S2. Correlation matrix between antioxidant capacity of GEMG and cytotoxicity against HCC cell lines.

	FRAP	ABTS	Hep3B	HepG2	Huh-7	PLC/PRF/5
**FRAP**	1	0.52615	-0.69688	-0.95066	-0.78775	-0.97755
**ABTS**	0.52615	1	-0.97655	-0.76401	-0.93831	-0.33516
**Hep3B**	-0.69688	-0.97655	1	0.88499	0.99075	0.53013
**HepG2**	-0.95066	-0.76401	0.88499	1	0.93999	0.86395
**Huh-7**	-0.78775	-0.93831	0.99075	0.93999	1	0.64028
**PLC/PRF/5**	-0.97755	-0.33516	0.53013	0.86395	0.64028	1

The data are expressed as the correlation coefficient, r. An r value close to +1 or -1 indicates a strong relationship, while a value near 0 indicates a weak or no relationship.

Table S3. Correlation matrix between antioxidant capacity of GEMM and cytotoxicity against HCC cell lines.

	FRAP	ABTS	Hep3B	HepG2	Huh-7	PLC/PRF/5
**FRAP**	1	0.97421	-0.67685	-0.022685	0.52554	0.92908
**ABTS**	0.97421	1	-0.8255	0.2035	0.70397	0.98858
**Hep3B**	-0.67685	-0.8255	1	-0.72058	-0.98198	-0.90113
**HepG2**	-0.022685	0.2035	-0.72058	1	0.83863	0.34871
**Huh-7**	0.52554	0.70397	-0.98198	0.83863	1	0.80296
**PLC/PRF/5**	0.92908	0.98858	-0.90113	0.34871	0.80296	1

The data are expressed as the correlation coefficient, r. An r value close to +1 or -1 indicates a strong relationship, while a value near 0 indicates a weak or no relationship.
